# Challenges and opportunities in flexible, stretchable and morphable bio-interfaced technologies

**DOI:** 10.1093/nsr/nwac016

**Published:** 2022-01-29

**Authors:** Abraham Vázquez-Guardado, Yiyuan Yang, John A Rogers

**Affiliations:** Center for Bio-Integrated Electronics, Northwestern University, USA; Querrey Simpson Institute for Bioelectronics, Northwestern University, USA; Department of Mechanical Engineering, Northwestern University, USA; Center for Bio-Integrated Electronics, Northwestern University, USA; Querrey Simpson Institute for Bioelectronics, Northwestern University, USA; Department of Mechanical Engineering, Northwestern University, USA; Department of Materials Science and Engineering, Northwestern University, USA; Department of Biomedical Engineering, Northwestern University, USA; Department of Neurological Surgery, Feinberg School of Medicine, Northwestern University, USA

Living organisms operate on the basis of dynamic biochemical processes elicited by a rich diversity of endogenous and exogenous stimuli. For higher-level forms of life, the result manifests as complex patterns of behavior and ultimately in the form of intelligence and consciousness. Emerging classes of biocompatible electronic interfaces support expanding possibilities in bidirectional communication. Examples include innovative interfaces that provide functional access to the central and peripheral nervous systems, vital organs and muscle tissues, as the basis of control (excitatory or inhibitory electrical stimuli (Fig. 1A-i)) and feedback (electrophysiology recordings (Fig. 1Ai–iii)) mechanisms, linked to implanted or externalized hardware and/or software systems for data collection and analytics. Potential applications in humans span stimulators for treating neurological disorders or chronic pain, to intraoperative devices for surgical uses or diagnostics. Furthermore, bidirectional interfacing provides opportunities in closed-loop operation for autonomous real-time control of biochemical processes (Fig. 1A-ii) relevant for the treatment and diagnosis of diseases or for brain–machine interfaces.

## CHALLENGES ON BIO-INTEGRATION

**Figure 1.**
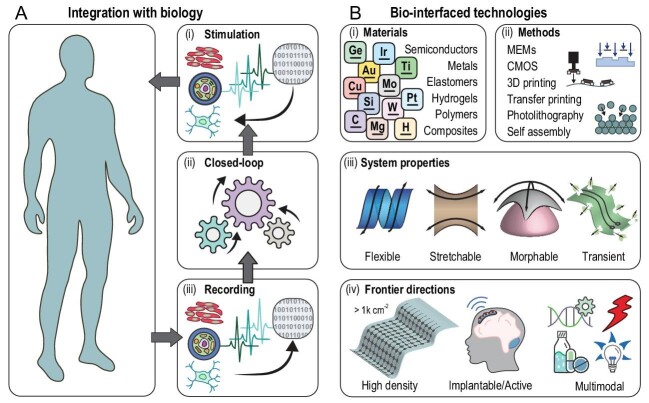
Challenges and opportunities in biological interfaces. (A) Recently developed biological interfaces offer promising applications in bidirectional communication with living organisms. Advanced implementations enable stimulation (i) and recording (iii) of cells with potential applications in closed-loop control (ii). (B) Such technologies originate from emerging classes of materials that combine excellent electrical and mechanical properties (i) and sophisticated fabrication methods (ii) that integrate these heterogeneous materials into flexible, stretchable, morphable and transient forms (iii) that enable their application not only in traditional electrical interfaces and fully implantable constructs, but also in multimodal systems for non-electrical stimulation and sensing (iv).

Operational time frames for such implantable biomedical devices depend on the scope of applications. Possibilities range from hours for acute intraoperative electrophysiology, clinical diagnostics and research applications, to patient lifetimes for chronic deep brain or peripheral nerve stimulation therapies. Traditional biomedical interfaces formed as arrays of penetrating probes made of common electrical materials (Fig. 1B-i) show excellent performance when used in acute modalities and even in certain long-term instances [1]. However, the poor mechanical compliance of these materials (silicon: ∼150 GPa, platinum: ∼160 GPa, tungsten: ∼200 GPa) with respect to soft tissues (brain and spinal cord tissues: 0.1–10 kPa, muscle tissue: ∼10–300 kPa) can elicit foreign-body responses, an intrinsic self-protecting biological process, that is further exacerbated by constant motions at device/tissue interfaces associated with natural motions and/or pulsations of the circulatory system [2]. Research and engineering efforts in biomedical interfaces that address this limitation use strategies in the form of polymeric materials with artificially enhanced electronic properties (Fig. 1B-i) [3] or exploiting heterogeneous integration between advanced electronic components [4]. The results are flexible, stretchable and even morphable interfaces (FSMIs; Fig. 1B-iii) with excellent biocompatibility and, in some cases, electronic perf ormance.

## FLEXIBLE, STRETCHABLE AND MORPHABLE INTERFACES

Realizing these FSMIs requires the use of diverse fabrication techniques (Fig. 1B-ii) including not only those well established and widely employed in the semiconductor industry, but also more recent advanced schemes in 3D printing, transfer printing and guided self-assembly [5]. Often, polymeric substrates (polyimide: ∼2.5 GPa, parylene: ∼2.1 GPa, elastomers: ∼0.4–1.2 MPa, hydrogels: ∼30 kPa) serve as the basis for thin probes, filamentary mesh constructs, nerve cuffs or flexible sheets with low stiffness (<10^–2^–10^–1^ N/m) to accommodate natural motions of the surrounding tissues [6]. Doping additives, surface treatment or micro/nano patterning the electrodes further enable necessary electrical characteristics at the biotic/abiotic interface, as defined by key metrics such as the interface impedance (Z < 0.3 Ωcm^2^ @ 1 kHz) and the charge injection capacity (CIC > 0.5 μC/cm^2^) for applications in high-fidelity recording (signal-to-noise ratio: SNR > 5) and efficient stimulation, respectively [1]. Ultimately, functional materials, such as conductive hydrogels and polymers with tissue-like mechanical properties, that exploit both ionic and electronic mechanisms of electrical conduction represent promising material candidates for bio-interfaced technologies [3].

An important frontier for research focuses on improving the spatio-temporal resolution, increasing the bandwidth of information and surface-area coverage, and in some cases establishing 3D interfaces (Fig. 1B-iv). Goals in spatio-temporal resolution include high bandwidth, >10 kHz per channel, and high-density ∼250 × 10^3^ electrodes/cm^2^ (transversal) or 500 electrodes/cm (longitudinal) recordings, with the ability to discriminate fast action potentials (∼1–2 ms) at a spatial resolution corresponding to single neurons (spacings ∼20 μm) [7]. Reaching these scales will enable exquisite mapping of neural dynamics and precise excitation of independent cells; however, it also demands advances not only in methods for micro/nanofabrication, in techniques for high-performance flexible electronics and in approaches for 3D architectures, but also in the development of materials that allow integration of electrode arrays that simultaneously reach the densities, coverages, impedances and CICs mentioned previously. Interesting options include conducting hydrogels and polymers with photolithographic processing capabilities to embed high-density electrodes [8].

**Figure 2.**
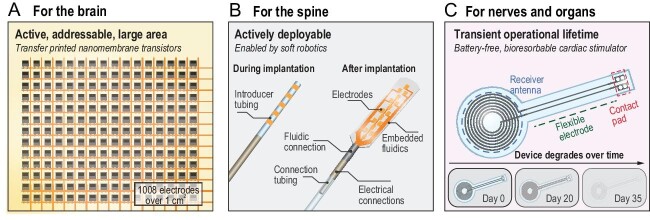
Examples of advanced biological interfaces. (A) Flexible, large-area, transfer-printed nanomembrane transistors for actively addressable platforms designed to map electrophysiological activity across the visual cortex of non-human primate models. The interface includes a thin polymetric substrate (∼7.5 μm) and 1008 actively multiplexed electrodes (195 × 270 μm^2^ size, 290 μm average inter-electrode spacing, 42 kΩ cm^2^ impedance) formed by transfer-printed nanomembrane silicon transistors. The image is reproduced with permission of Ref [9]. (B) Syringe-implantable paddle-type electrodes, enabled by soft robotics, that roll up inside hollow needles (∼2 mm in diameter) to facilitate minimally invasive implantation. Unfurling into flat geometries follows from fluidic actuation, to allow soft interfaces to human spinal cord tissues. This device contains lithographically defined metal electrodes and interconnections (Ti (10 nm)/Au (100 nm)) encapsulated with parylene (∼1 μm), supported by a bilayer silicone structure (∼30–60 μm) that surrounds a stylet tube. Two bismuth powder-infused silicone cords, located along the flank of the device, serve as X-ray opaque markers. The image is reproduced with permission of Ref. [11]. (C) Bioresorbable, battery-free cardiac simulator with controlled rate of dissolution in biofluids. This device contains a wireless power-receiver system composed of an inductive coil (W (∼700 nm)/Mg (∼50 μm)) and PIN diode (Si nanomembrane (320 nm)) and a dielectric interlayer (PLGA (50 μm)), as the control module to deliver pacing pulses to cardiac tissue via exposed contact pads (W (∼700 nm)/Mg (∼50 μm)) connected with flexible electrodes (W (∼700 nm)/Mg (∼50 μm)). The device is encapsulated with a bioresorbable layer (PLGA, 100 μm). Image is reproduced with permission of Ref. [13].

Active electronics for distributed signal multiplexing, amplification and/or conditioning, with additional potential for wireless communications, are required to fully exploit advances in FSMIs (Fig. 1B-iv). Sophisticated modular active silicon micro-components, such as application-specific integrated circuits or high-quality thin-film transistors, can be fabricated in large volumes with the highest-quality standards and design rules set by the display and integrated circuit industries. For instance, single or arrayed configuration of thin-film transistors, which are integral components in active interfaces, can be designed to serve as basic modular building blocks of active FSMIs. Transfer printing techniques that allow high-throughput assembly of these micro-components on a large scale onto delicate substrates support promising routes to increased modularity, functionality and spatio-temporal resolution. Recent progress includes demonstrations of transfer-printed nanomembrane silicon transistors as the basis of thin, flexible micro-electrocorticography (μ-ECoG) devices that include 1008 capacitively coupled and actively multiplexed electrodes on polymer substrates as thin as 47.5 μm and with areas of ∼1 cm^2^ as interfaces across the visual cortex of non-human primate models (Fig. 2A) [9].

## CHALLENGES IN SYSTEM-LEVEL INTEGRATION

Fully implantable platforms represent an important goal (Fig. 1B-iv), with additional challenges for bio-integration [2]. In some cases, foreign-body responses degrade the active sensing/recording elements, fibrous tissue growth isolates the devices and/or biofluids disrupt electronic operation. Material-level compatibility, shape-matching forms, low-modulus mechanics and miniaturized data acquisition, communication and power systems must all be considered collectively to address bio-integration challenges (Fig. 1B). Methods in wireless power transfer and/or in energy harvesting from biophysical or biochemical processes are of particular interest, to eliminate batteries or significantly reduce requirements in their storage capacities. Trends in low-power electronics and radio-communication hardware for consumer electronic devices are highly relevant in this context, provided that the FSMIs are designed to adapt or align with such technologies. These systems can be configured to support data transmission and bidirectional communication, including sensors (temperature, strain gauges, photometry, electrophysiology, etc.) for closed-loop operation. Architectural designs that centralize the electronic and power modules permit their physical separation from the actuators and/or sensors located in or on soft, delicate neural tissues via flexible and mechanically compliant interconnects [10]. Such form factors and modular layouts are rapidly emerging as well-accepted strategies in system construction.

## GENETIC STRATEGIES FOR MULTIMODAL BIO-INTERFACED TECHNOLOGIES

An important set of strategies that bypasses the need for electrical interfaces, but still leverages many of the essential materials and device approaches outlined previously (Fig. 1B-iv), use genetically modified cells that respond to light (optogenetics), exogenous pharmacology compounds (pharmacogenetics) or those that transduce intracellular concentration of biochemicals (e.g. calcium) into readable optical signals (photometry). Multimodal FSMIs follow naturally from progress in the various directions outlined above. For example, separately controlled optogenetic and pharmacological mechanisms for stimulation can be combined into integrated platforms that also incorporate electrodes for electrophysiological recording. Such systems will establish exciting possibilities in biomedical research, with promising potential for future therapeutic application in humans.

## FUTURE DIRECTIONS

Research opportunities in the science and engineering aspects of these FSMI technologies further considers minimally invasive approaches for surgical implantation and, in some cases, for extraction after a period of need.

Systems that deploy or distribute themselves automatically or upon an external trigger after delivery through a syringe or catheter are promising. Examples in this context, inspired by recent advances in soft robotics, include syringe-implantable paddle-type electrodes that roll up to allow implantation through hollow needles, to subsequently unfold into flat geometries that are integrated on the surfaces of neural tissues via fluidic actuation (Fig. 2B) [11].

Challenges in device extraction can be eliminated entirely through use of emerging capabilities in transient or bioresorbable electronic, optoelectronic, microfluidic and/or microelectromechanical systems. Here, polymer materials (poly(vinyl alcohol); polyvinylpyrrolidone; polylacticcoglycolic acid; polylactic acid; polycaprolactone; poly(1,8-octanediol-*co*-citrate, polyurethane)) in conjunction with naturally degrading metals (magnesium; zinc; iron; tungsten; molybdenum), semiconductors (silicon; germanium) or their alloys provide enabling materials. The intrinsic structural forms (material properties and device-form factors) and extrinsic biochemical environments (pH, light and temperature) collectively determine the rates of resorption. Recent examples are in bioresorbable sensors (pressure/temperature sensing in the intracranial space) [12] and electrical stimulators (wireless temporary pacing on the cardiac surface) (Fig. 2C)) [13].

## CONCLUSIONS AND OUTLOOK

Research in materials science, chemistry and applied physics will continue to form the foundations for innovative work in bio-interfaced technologies, where the scope of application defines a trade-off between system biocompatibility and functionality. Important trends are in multimodal function in both neuromodulation and biophysical/biochemical sensing, with opportunities in adaptive operation using feedback loops generically inspired by those that sustain living organisms. Considering these various possibilities in permanent and transient interfaces in the context of full 3D integration in a minimally invasive manner might represent some of the most daunting and most compelling directions. The outcomes derived from these activities have the potential to resonate into transformative developments that will aid in our understanding of physiological disabilities and neurological and medical disorders, and enable development of advanced therapeutic and diagnostic practices. The most impactful results will follow from the creation of bio-interfaced technologies that improve our quality of life and extend our lifetimes.

***Conflict of interest statement***. J.A.R. is cofounder in a company, NeuroLux Inc., that offers optogenetic technology products to the neuroscience community.
